# MicroRNA meta-signature of oral cancer: evidence from a meta-analysis

**DOI:** 10.1080/03009734.2018.1439551

**Published:** 2018-02-26

**Authors:** Katarina Zeljic, Ivan Jovanovic, Jasmina Jovanovic, Zvonko Magic, Aleksandra Stankovic, Gordana Supic

**Affiliations:** a Faculty of Biology, University of Belgrade, Belgrade, Serbia; b Laboratory for Radiobiology and Molecular Genetics, University of Belgrade, Vinča Institute of Nuclear Sciences, Belgrade, Serbia; c Faculty of Mathematics, University of Belgrade, Belgrade, Serbia; d Faculty of Medicine, Military Medical Academy, University of Defence, Belgrade, Serbia; e Institute for Medical Research, Military Medical Academy, Belgrade, Serbia

**Keywords:** Enrichment analysis, meta-analysis, meta-signature, miRNA, oral cancer

## Abstract

**Aim:**

It was the aim of the study to identify commonly deregulated miRNAs in oral cancer patients by performing a meta-analysis of previously published miRNA expression profiles in cancer and matched normal non-cancerous tissue in such patients.

**Material and methods:**

Meta-analysis included seven independent studies analyzed by a vote-counting method followed by bioinformatic enrichment analysis.

**Results:**

Amongst seven independent studies included in the meta-analysis, 20 miRNAs were found to be deregulated in oral cancer when compared with non-cancerous tissue. Eleven miRNAs were consistently up-regulated in three or more studies (miR-21-5p, miR-31-5p, miR-135b-5p, miR-31-3p, miR-93-5p, miR-34b-5p, miR-424-5p, miR-18a-5p, miR-455-3p, miR-450a-5p, miR-21-3p), and nine were down-regulated (miR-139-5p, miR-30a-3p, miR-376c-3p, miR-885-5p, miR-375, miR-486-5p, miR-411-5p, miR-133a-3p, miR-30a-5p). The meta-signature of identified miRNAs was functionally characterized by KEGG enrichment analysis. Twenty-four KEGG pathways were significantly enriched, and TGF-beta signaling was the most enriched signaling pathway. The highest number of meta-signature miRNAs was involved in the sphingolipid signaling pathway. Natural killer cell-mediated cytotoxicity was the pathway with most genes regulated by identified miRNAs. The rest of the enriched pathways in our miRNA list describe different malignancies and signaling.

**Conclusions:**

The identified miRNA meta-signature might be considered as a potential battery of biomarkers when distinguishing oral cancer tissue from normal, non-cancerous tissue. Further mechanistic studies are warranted in order to confirm and fully elucidate the role of deregulated miRNAs in oral cancer.

## Introduction

Oral cancer is the most common type of head and neck cancers, characterized by high mortality rates, low long-term survival, and an increasing incidence among younger people (1). As the complexity of oral cancer is still challenging contemporary medicine, further molecular characterization of this cancer type is required. Identification of novel accurate molecular biomarkers will facilitate early detection and confirmation of the oral cancer diagnosis at the molecular level.

MicroRNA (miRNA) is a class of small non-coding RNA molecules which act as negative regulators of target gene expression at the post-transcriptional level (2). MicroRNAs induce gene silencing by binding to the 3′ untranslated region (3′-UTR) of the target mRNA, which leads to either mRNA degradation or translational repression (2). Almost all key cellular processes, such as proliferation, development, differentiation, and apoptosis, are regulated by miRNAs (2). Therefore, it is not surprising that altered expression of particular miRNAs has been associated with occurrence of cancer, including oral cancer (3,4).

Identification of deregulated miRNAs in oral cancer is important in the search for new sensitive molecular biomarkers with diagnostic, predictive, and prognostic value (3–5). Nowadays, the development of high-throughput technologies has enabled the identification of differentially expressed miRNAs between cancer and non-cancerous tissue samples on a large scale. However, the technologies used in different studies are diverse, and the obtained results are not entirely consistent. Therefore, our aim was to identify commonly deregulated miRNAs in oral cancer and characterize their biological meaning by performing a comprehensive meta-analysis of previously published miRNA expression profiling studies, followed by bioinformatic enrichment analysis.

## Material and methods

### Search strategy and study inclusion/exclusion criteria

Literature searching (PubMed) was carried out by using different combinations of keywords: miRNA, microRNA, miR, profile, profiling, signature, oral, cancer, tumour, tumor, carcinoma, squamous cell carcinoma. A last search was done on 31 January 2017. The meta-analysis was conducted to review published studies that compared the miRNA expression profiles in oral cancer tissues with those in normal, matched oral tissues. Therefore, we did not include miRNA expression data from public databases such as TCGA (The Cancer Genome Atlas) and GEO (Gene Expression Omnibus). The following inclusion criteria were considered: (1) original articles published in English with full text available; (2) miRNA expression profiling in oral cancer specimens and matched non-cancerous counterparts of the same patients; (3) studies with reported lists of differentially expressed miRNAs and cut-off criteria. Papers in other languages, review articles, studies conducted on cell lines, analyzed expression of candidate miRNAs, oral cancer tissue and healthy tissue collected from different individuals, studies where different patient’s biological samples (such as blood, saliva, or serum) were used were excluded from further analysis. Data provided in the article’s supplementary material were also taken into consideration. Studies with no reported lists of differentially expressed miRNAs, or data that had only been shown on the heatmap, where further information could not be obtained, were excluded from the meta-analysis as well.

### Meta-analysis of commonly deregulated miRNAs in oral cancer

From studies included in the meta-analysis, lists of differentially expressed miRNAs and their regulated directions in oral cancer were collected. Annotations of differentially expressed miRNAs in the different studies were harmonized according to the miRBase database (www.mirbase.org) (release 21 June 2014). If the miRNA strand was not reported, dominant or more abundant forms from the miRBase were used if applicable (6).

Meta-analysis was performed by means of a vote-counting method, which considers the total number of studies reporting consistently differentially expressed miRNAs (7,8). Fold change was not reported in all included studies. Therefore, this criterion was excluded in the miRNA meta-signature identification by vote-counting strategy. Seven studies were analyzed, and miRNAs reported as differentially expressed in at least three or more studies were considered as commonly deregulated.

The analyses were performed using R programming language and software environment for statistical computing and graphics. R scripts are available in the Supplement material S1 (available online).

### Bioinformatic analysis

In order to access regulatory roles and identify molecular pathways controlled by commonly deregulated miRNAs in oral cancer, online open software DIANA-miRPath v3.0 (www.microrna.gr/miRPathv3) was used (9). The algorithm microT CDS v5.0 was used as prediction algorithm of miRNA–gene interactions. DIANA-miRPath v3.0 uses meta-analysis statistics for the assessment of combined miRNA action. The meta-analysis algorithm enables the identification of pathways controlled by multiple miRNAs by examining each miRNA individually and subsequently combining the result probabilities and test statistics (9). Functional analysis of commonly deregulated miRNAs was done for Kyoto Encyclopedia of Genes and Genomes (KEGG) pathways and Gene Ontology (GO) biological process terms (9). Results were merged into pathway and category union for KEGG and GO analysis, depicting 1–the probability that the examined functional category is significantly enriched with gene targets of at least one selected miRNA. For the enrichment analysis method, unbiased empirical distributions (10) with a microT threshold of 0.7 and false discovery rate (FDR) correction were used. Molecular pathways were considered significantly enriched when *p* < 0.05. Heatmaps of significantly enriched pathways were constructed using the DIANA-miRPath v3.0 tool (9).

## Results

### Overview of the studies included in the meta-analysis

By searching the PubMed database using different combinations of the previously mentioned keywords, avoiding duplications and review papers, and after careful consideration of all collected studies, seven studies met all inclusion criteria and were considered for further analysis (11–17). Details of the studies included in the meta-analysis are presented in Table 1. For the identification of deregulated miRNAs in oral cancer and matched normal tissue, different platforms and cut-off values were utilized (Table 1). From studies which met the required inclusion criteria, lists of differentially expressed miRNAs were collected (Supplementary material S2, available online). A total of 184 oral cancer samples and 162 matched adjacent normal tissue samples were analyzed. Altogether in the studies, 215 distinct miRNAs were identified to be reported as differentially expressed, after harmonization of annotations. In the list of all identified miRNAs, 93 miRNAs showed up-regulation in studies where identified, 111 miRNAs showed down-regulation, and 11 miRNAs showed inconsistent regulation (Supplementary material S3, available online).

**Table 1.** Details of the included studies.

Study (reference)	Number of tissue samples (cases/control)^a^	Differentially expressed miRNA			Cut-off criteria	Platform	Country
		Total	Up-regulated	Down-regulated			
Ganci et al., 2016 (11)	76 (38/38)	78	59	19	FDR <0.06 *p* < 0.01 FC >1	Agilent platform Human miRNA microarray (V2)	Italy, Europe
Manikandan et al., 2016 (12)	58 (29/29)	39	15	24	SD >1 FC >1 *p* < 0.05	miRCURY LNA™ microRNA array (Exiqon)	India, Asia
Shiah et al., 2014 (13)	80 (40/40)	84	32	52	FC >2 *p* < 0.05	Human v2 microRNA expression BeadChips (Illumina)	Taiwan, Asia
Soga et al., 2013 (14)	36 (29/7)	23	12	11	FC >4	TaqMan Low Density Array (Human microRNA Panel v2.0)	Japan, Asia
Shi et al., 2015 (15)	4 (2/2)	38	31	7	FC >2	RNA Seq (Illumina HiSeq 2000)	China, Asia
Fukumoto et al., 2015 (16)	72 (36/36)	42	NR	42	*p* < 0.05	TaqMan Low Density Array (Human microRNA Panel v2.0)	Japan, Asia
Chen et al., 2017 (17)	20 (10/10)	12	7	5	*p* < 0.05 *p* < 0.01	TaqMan Low Density Array (TLDA v1.0)	USA

a Oral cancer tissue specimens/matched adjacent non-cancerous tissue from the same individual.

FC: fold change; FDR: false discovery rate; NR: not reported; SD: standard deviation.

### MicroRNA meta-signature of oral cancer

After harmonization of annotations, miRNAs found to be deregulated in at least three studies were considered as commonly deregulated (Table 2). Among 20 commonly deregulated miRNAs in oral cancer compared to matched non-cancerous tissue, 11 were consistently up-regulated (miR-21-5p, miR-31-5p, miR-135b-5p, miR-31-3p, miR-93-5p, miR-34b-5p, miR-424-5p, miR-18a-5p, miR-455-3p, miR-450a-5p, miR-21-3p), while nine were consistently down-regulated (miR-139-5p, miR-30a-3p, miR-376c-3p, miR-885-5p, miR-375, miR-486-5p, miR-411-5p, miR-133a-3p, miR-30a-5p). Throughout our study miR-223-3p, miR-203-3p, and miR-17-5p also passed the identification threshold, but showed different regulated directions (Table 2). Therefore, for further analysis only miRNAs with consistent regulated direction in oral cancer were considered in the oral cancer meta-signature, while miR-223-3p, miR-203-3p, and miR-17-5p were excluded due to expression inconsistencies. Two of the most commonly up-regulated miRNAs in oral cancer, identified in five out of seven studies, were miR-21-5p and miR-31-5p. In a signature of consistently reported up-regulated miRNAs, miR-31-3p and miR-135b-5p were reported in four studies. Four studies also showed down-regulation of miR-139-5p, miR-30a-3p, and miR-376c-3p in oral cancer when compared with non-cancerous tissue. The rest of the deregulated miRNAs, included in the oral cancer meta-signature, were reported in three studies out of seven (Table 2).

**Table 2.** List of commonly deregulated miRNAs in oral cancer compared with matched non-cancerous tissue among different studies.

Deregulated miRNAs	Common: studies (*n*)	Up-regulated: studies (*n*)	Down-regulated: studies (*n*)	Present in study	Samples (*n*)^a^
miR-21-5p	5	5	0	Ganci et al., 2016; Soga et al., 2013; Shi et al., 2015; Chen et al., 2017; Manikandan et al., 2016	176
miR-31-5p	5	5	0	Ganci et al., 2016; Soga et al., 2013; Chen et al., 2017; Manikandan et al., 2016; Shiah et al., 2014	252
miR-139-5p	4	0	4	Soga et al., 2013; Fukumoto et al., 2015; Chen et al., 2017; Shiah et al., 2014	208
miR-30a-3p	4	0	4	Soga et al., 2013; Shi et al., 2015; Fukumoto et al., 2015; Shiah et al., 2014	192
miR-376c-3p	4	0	4	Ganci et al., 2016; Soga et al., 2013; Fukumoto et al., 2015; Shiah et al., 2014	264
miR-223-3p^b^	4	3	1	Ganci et al., 2016; Soga et al., 2013; Chen et al., 2017; Manikandan et al., 2016	190
miR-135b-5p	4	4	0	Ganci et al., 2016; Soga et al., 2013; Shi et al., 2015; Shiah et al., 2014	196
miR-31-3p	4	4	0	Ganci et al., 2016; Soga et al., 2013; Manikandan et al., 2016; Shiah et al., 2014	250
miR-885-5p	3	0	3	Shi et al., 2015; Fukumoto et al., 2015; Shiah et al., 2014	156
miR-375	3	0	3	Shi et al., 2015; Fukumoto et al., 2015; Shiah et al., 2014	156
miR-486-5p	3	0	3	Soga et al., 2013; Chen et al., 2017; Shiah et al., 2014	140
miR-411-5p	3	0	3	Soga et al., 2013; Fukumoto et al., 2015; Shiah et al., 2014	188
miR-203-3p^b^	3	2	1	Soga et al., 2013; Shi et al., 2015; Manikandan et al., 2016	98
miR-133a-3p	3	0	3	Ganci et al., 2016; Soga et al., 2013; Shiah et al., 2014	192
miR-30a-5p	3	0	3	Ganci et al., 2016; Shi et al., 2015; Shiah et al., 2014	160
miR-17-5p^b^	3	2	1	Ganci et al., 2016; Shi et al., 2015; Manikandan et al., 2016	138
miR-93-5p	3	3	0	Ganci et al., 2016; Soga et al., 2013; Shi et al., 2015	116
miR-34b-5p	3	3	0	Ganci et al., 2016; Shi et al., 2015; Shiah et al., 2014	160
miR-424-5p	3	3	0	Ganci et al., 2016; Shi et al., 2015; Shiah et al., 2014	160
miR-18a-5p	3	3	0	Ganci et al., 2016; Shi et al., 2015; Shiah et al., 2014	160
miR-455-3p	3	3	0	Ganci et al., 2016; Shi et al., 2015; Shiah et al., 2014	160
miR-450a-5p	3	3	0	Ganci et al., 2016; Shi et al., 2015; Shiah et al., 2014	160
miR-21-3p	3	3	0	Ganci et al., 2016; Shi et al., 2015; Shiah et al., 2014	160

a Number of tissue samples tested across the studies.

b Due to inconsistencies in expression among studies, miRNAs were not further considered as commonly deregulated in oral cancer compared with matched non-cancerous tissue.

### Functional annotation of commonly deregulated miRNAs in oral cancer

**Figure 1.**
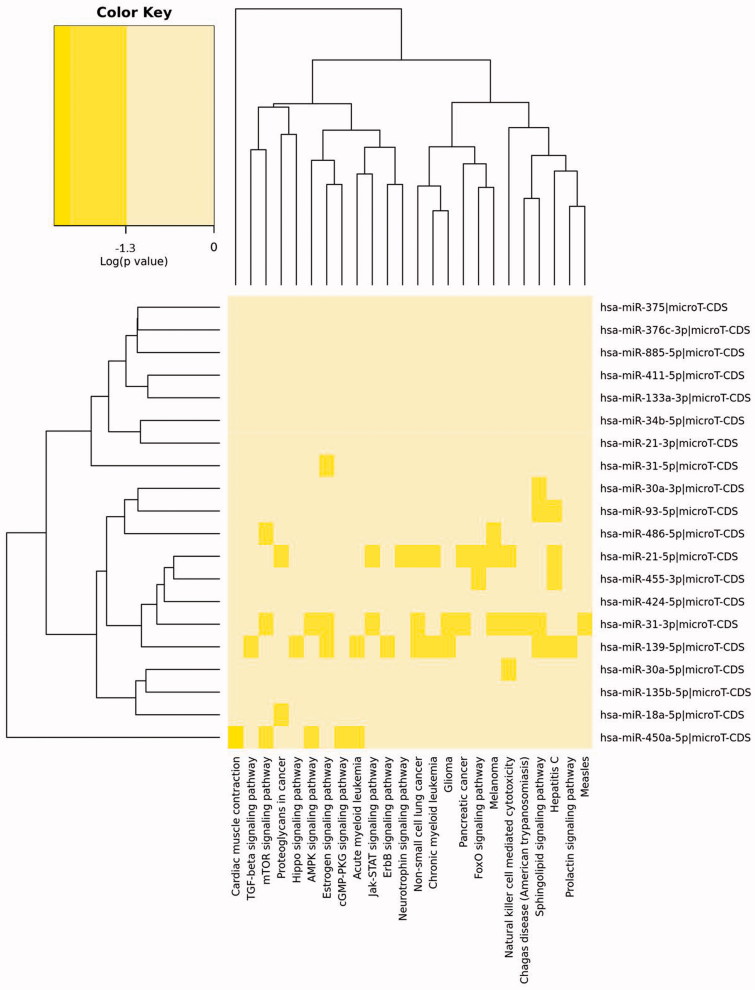
Heatmap of KEGG pathways enriched in oral cancer miRNA meta-signature. The heatmap depicts the enrichment level of KEGG pathways in miRNAs commonly deregulated in oral cancer (microT CDS v5.0 was used for target prediction, *p* value threshold 0.05, microT threshold 0.7, and enrichment analysis method: unbiased empirical distributions). Intensity of colors represents the log (*p* value).

For further functional characterization the identified miRNA meta-signature was the subject of GO and KEGG enrichment analysis. We have noticed that GO enrichment using unbiased empirical distributions approach gave insufficient enrichment results, in contrast to the Fisher exact test (data not shown). However, it was recently pointed out that miRNA enrichment analysis using the Fisher test introduces bias (10), so we decided to proceed with the knowledge acquired by unbiased empirical distributions method. Twenty-four KEGG pathways were significantly enriched in the oral cancer miRNA meta-signature (Table 3, Figure 1). Pathways enriched in our miRNA list are coupled to different malignancies. Also, there was a plethora of signaling pathways. TGF-beta was the most enriched signaling pathway, while the highest number of meta-signature miRNAs regulate genes involved in the sphingolipid signaling pathway. The pathway with the highest number of genes regulated by identified miRNAs was the natural killer cell (NK)-mediated cytotoxicity (Table 3, Figure 1).

**Table 3.** List of KEGG pathways enriched in oral cancer miRNA meta-signature.

KEGG pathway	*p* value^a^	Genes (*n*)	MiRNAs (*n*)
Acute myeloid leukemia	0.006443	3	2
TGF-beta signaling pathway	0.011516	4	1
Hepatitis C	0.013149	19	4
Proteoglycans in cancer	0.015179	28	2
Non-small cell lung cancer	0.016204	9	3
Estrogen signaling pathway	0.019171	18	3
Pancreatic cancer	0.022215	10	2
Cardiac muscle contraction	0.023825	2	1
Sphingolipid signaling pathway	0.02472	17	4
AMPK signaling pathway	0.026242	8	2
ErbB signaling pathway	0.026337	3	1
Natural killer cell-mediated cytotoxicity	0.028397	29	3
Neurotrophin signaling pathway	0.032315	12	1
mTOR signaling pathway	0.032902	10	3
Chronic myeloid leukemia	0.033489	9	2
Hippo signaling pathway	0.033673	5	1
FoxO signaling pathway	0.035991	18	2
cGMP-PKG signaling pathway	0.036768	3	1
Melanoma	0.03704	18	3
Jak-STAT signaling pathway	0.037747	14	2
Prolactin signaling pathway	0.040764	4	1
Glioma	0.042782	6	2
Chagas disease (American trypanosomiasis)	0.04822	6	1
Measles	0.049572	4	1

a *p* < 0.05 was considered as statistically significant.

## Discussion

The fast development of high-throughput technologies has enabled easier identification of possible molecular biomarkers for different pathologies, including cancer. However, due to differently used platforms, sample sizes, data analysis, and interpretation, a high level of inconsistency among studies exists (11–17). Therefore, the need for integration of results from different studies through meta-analyses is justified.

Results of deregulated miRNAs reported in the studies included in the current meta-analysis are inconsistent. Numerous factors can contribute to this inconsistency, for instance, small effect sizes, sample preparation and storage, clinical characteristics of the patients, normalization of obtained data and data analysis, and different cut-off criteria for identification of differentially expressed miRNAs. These noticed inconsistencies among studies might emerge from differences in used platforms and types and numbers of probes as well. Even intra-tumor heterogeneity of miRNA expression (18,19) as well as molecular changes related to cancerogenesis in morphologically normal tissue (20) could introduce the inconsistency.

Our results regarding *mir-21*, *mir-31*, *mir-133*, and *mir-139* deregulation in oral cancer are in line with findings reported in a recently published meta-analysis (21). Existing non-overlapping miRNAs between that and our study can be explained with more rigorous criteria applied in the selection process of our study. The finding of an up-regulation of *mir-21* was not a surprise, since *mir-21* is considered as an oncomir and therefore is highly expressed in various cancer types (22). *Mir-21* has been considered to be a potential biomarker for tongue cancer detection (23) and progression and survival (24). Increased *mir-21* expression is associated with poor survival of patients with head and neck cancer according to recently conducted meta-analysis (25). It has also been shown that miR-21-3p is overexpressed in oral cancer and that it plays an important role in metastasis (26). Likewise, the up-regulation of *mir-31* in oral cancer is probably a result of epidermal growth factor receptor (*EGFR*) activation (27). Increased expression of *mir-31* was also noticed in early stages of oral cancer with no detected metastasis (28). Up-regulation of *mir-31* was proposed as a suitable biomarker of increased malignant transformation of oral, potentially malignant disorders (29). Down-regulation of miR-133a-3p was reported in oral cancer as well as in oral cancer cell lines (30). Oral cancer proliferation and invasion can be suppressed by miR-133a-3p over-expression and collagen type I alpha 1 chain gene (*COL1A1*) direct targeting (30). Decreased expression of miR-139-5p has been reported as a potential saliva biomarker for tongue cancer detection (31).

The activity of other meta-signature miRNAs was also examined in oral cancer. In a study investigating miRNA expression in oral cancer patients with and without metastasis, *mir-135b* was suggested to be a promising down-regulated biomarker of oral cancer progression (32). It was found that down-regulated miR-376c-3p suppresses the cancer phenotype by targeting homeobox B7 (*HOXB7*) in oral cancer tissue as well as in cell lines (33). In addition, in head and neck cancer, down-regulated miR-376c-3p leads to runt-related transcription factor 2 gene (*RUNX2*) deregulation and is associated with lymph node metastasis (34). Poor prognosis of head and neck cancer patients was associated with up-regulated expression of *mir-93* (35) and *mir-18a* (25). *Mir-34b* has been found to be one of the consistently up-regulated miRNAs in head and neck cancer in a majority of studies as well (36). A recent study reported that in oral cancer miR-424-5p directly targets supressor of cytokine signaling gene (*SOCS2*) and modulates STAT5 signaling, expression of matrix metalloproteinase, cell migration, and invasion (37). It has been reported that miR-455-3p up-regulation is controlled by the TGF-beta pathway, consequently down-regulating ubiquitin conjugating enzyme E2B gene (*UBE2B*), which results in oral cancer proliferation (38).

Recent studies have emphasized the utility of *mir-375* as a biomarker for detection of tongue cancer (23), malignant transformation of oral lesions (39), and late stages of oral cancer (28). *Mir-375* has been down-regulated in metastatic oral cancer cell lines compared with less metastatic cells, and *mir-375* induced cell migration and invasion by direct targeting the platelet-derived growth factor-A (*PDGF-A*) (40). *Mir-375* mediates the trichostatin-A-induced radiosensitization of tongue cancer (41), indicating a therapeutic potential of *mir-375.* In addition, down-regulation of plasma circulating *mir-375* and miR-486-5p has been associated with oral cancer recurrence (42). To the best of our knowledge, expression of *mir-30a* has not been studied in oral cancer. However, it has been reported that alcohol induces deregulation of *mir-30a* by increasing its expression in head and neck cancer (43).

It is well established that aberrant regulation of the signaling pathways is a prevalent theme in cancer. The most enriched signaling pathway is that of TGF-beta. It is involved in the regulation of crucial cellular processes of importance for cancerogenesis: cell growth, differentiation, apoptosis, and angiogenesis (44). The dual role of TGF-beta has been proposed in cancer formation—from protection from cancer progression in the early stages, to cancer promotion in later stages (44). Regarding enrichment analysis, the highest number of miRNAs regulate genes involved in the sphingolipid signaling pathway. Ceramide, sphingosine-1-phosphate, and their signaling cascade have been implicated in various aspects of oncogenesis and cancer progression (45). The main reason is that both lipids regulate the PI3K/AKT pathway, which represses apoptosis and autophagy (45). Sphingolipids also have an impact on cell cycle progression, telomerase function, cell migration, and stem cell biology (45). On the other hand, most of the genes regulated by miRNAs in our analysis belonged to the natural killer cell (NK)-mediated cytotoxicity. *In vitro* studies on human and other mammal cells, as well as *in vivo* studies in rodents, have suggested that NK cells target tumor cells (46). However, it has been suggested that tumors release exosomes which inhibit IL-2-induced NK cell cytotoxicity (47). It is known that exosomes contain miRNAs (48), which have the potential to regulate processes involved in NK cell homeostasis, survival, and turnover. As the cancerous and healthy tissues were obtained from the same patients, differentially expressed miRNAs might be involved in NK cytotoxicity modulation if transmitted by exosomes from cancer cells to NK cells via previously observed mechanisms (49).

When interpreting results obtained by the vote-counting method, some considerations have to be taken into account, keeping in mind different limitations and shortcomings (50). However, the vote-counting method has been recommended and is considered as a good alternative for identification of consistent gene expression (7,8), as well as a reliable strategy in searching for potential miRNA biomarkers in colorectal (51) and lung cancer (52).

To sum up, results reported in the current study can be helpful for more focused future confirmatory analysis of pre-selected miRNAs expression in oral cancer patients. Further validation and mechanistic studies of reported miRNA signature are highly warranted. It would also be interesting to perform more in-depth analysis of identified miRNA meta-signature in association with clinical data, such as patient survival, outcome, and metastasis.

## Supplementary Material

Supplemental data
